# A case of *de novo* neuroendocrine prostate cancer presented with elevated level of serum CEA carrying BRCA2 mutation: case report and literature review

**DOI:** 10.3389/fonc.2025.1508410

**Published:** 2025-01-24

**Authors:** Weizhe Han, Nihati Rexiati, Fang Yu, Yongzhi Wang, Yueli Tian, Jianyuan Wu, Gang Wang, Tao Liu, Zhonghua Yang

**Affiliations:** ^1^ Department of Urology, Zhongnan Hospital of Wuhan University, Wuhan, China; ^2^ Department of Pathology, Zhongnan Hospital of Wuhan University, Wuhan, China; ^3^ Department of Nuclear Medicine, Zhongnan Hospital of Wuhan University, Wuhan, China; ^4^ Clinical Trial Center, Zhongnan Hospital of Wuhan University, Wuhan, China; ^5^ Department of Biological Repositories, Human Genetic Resource Preservation Center of Hubei Province, Zhongnan Hospital of Wuhan University, Wuhan, China

**Keywords:** neuroendocrine prostate cancer (NEPC), carcinoembryonic antigen (CEA), homologous recombination repair (HRR), marker, prostate cancer

## Abstract

**Background:**

*De novo* neuroendocrine prostate cancer (NEPC) is a rare subtype of prostate cancer (PCa) and few markers are available for screening and monitoring. Potential circulating or fluid markers might facilitate early diagnosis thus improving prognosis of NEPC, especially for *de novo* NEPC.

**Case presentation:**

A man of 71-year was presented with elevated level of serum carcinoembryonic antigen (CEA) (1296.5 ng/ml) and normal PSA (0.47ng/ml). Gastrointestinal endoscopy showed no signs of gastric or colorectal cancer. Fluorodeoxyglucose positron emission tomography-computed tomography (FDG PET-CT) and prostate-specific membrane antigen PET-CT (PSMA PET-CT) indicated prostate cancer with metastases including pelvic lymph nodes, bone as well as lung metastases. Biopsy of prostate revealed mixed carcinoma including small cell neuroendocrine carcinoma (SCNEC) and adenocarcinoma (Gleason score of 4 + 5). Immunohistochemistry (IHC) staining and next generation sequencing demonstrated a strong expression of chromogranin A (CgA), synaptophysin (SYN) and CEA, and a germline mutation in BRCA2, respectively. After a prostatic massage, an increased level of CEA (137 ng/ml vs 5 ng/ml) was detected in urine. Olaparib, a Poly ADP-ribose polymerase inhibitor (PARPi), combined with androgen deprivation therapy (ADT) were administrated. FDG PET-CT indicated tumor regression in both quantity and size three months later, and CEA levels of serum and urine decreased to 23 ng/ml and 2.4 ng/ml 4 months later, respectively.

**Conclusion:**

This is the first report of a *de novo* NEPC presented with an elevated level of CEA, in which CEA was also detected in urine specimen post a prostatic massage. After a combination treatment of ADT for 3 months, levels of CEA in both serum and urine decreased sharply when tumor regressed radiologically. CEA might be a marker of screening and monitoring of NEPC.

## Introduction

Neuroendocrine prostate carcinoma (NEPC) is a rare subtype of prostate cancer (PCa), characterized by an extremely poorer prognosis, resistance to hormone therapy, rapid progression and visceral metastases (1). Treatment-induced NEPC (t-NEPC) can be detected in approximately 20% of metastatic castration-resistant prostate cancer (mCRPC) in response to hormonal therapy, especially androgen receptor pathway inhibitors (ARPI), such as abiraterone, apalutamide and enzalutamide. *De novo* NEPC is rather rarer, accounting for less than 1% of all PCa cases (2). It is more difficult to be diagnosed than t-NEPC since the latter always has a prior novel hormonal therapy for metastatic prostate cancer and an elevating level of neuroendocrine (NE) markers, such as neuron-specific enolase (NSE). Almost all cases are viscerally metastatic at diagnosis and few effective treatment options are available, thus median survival for metastatic NEPC was only about 10 months (1).

Immunohistochemically, these tumors can either express the conventional NE markers, including chromogranin (CgA) and synaptophysin (SYN), with or without CD56 expression, or even have complete lack of expression of any NE marker in about 10% of cases (3). These make NEPC a challenge for both the urologist and pathologist.

So, a potential fluid marker of high sensitivity, specificity and ease of detection, if available, might facilitate the early screening or diagnosis of NEPC, especially *de novo* NEPC and improve the prognosis of NEPC.

## Case presentation

**Figure 1:**
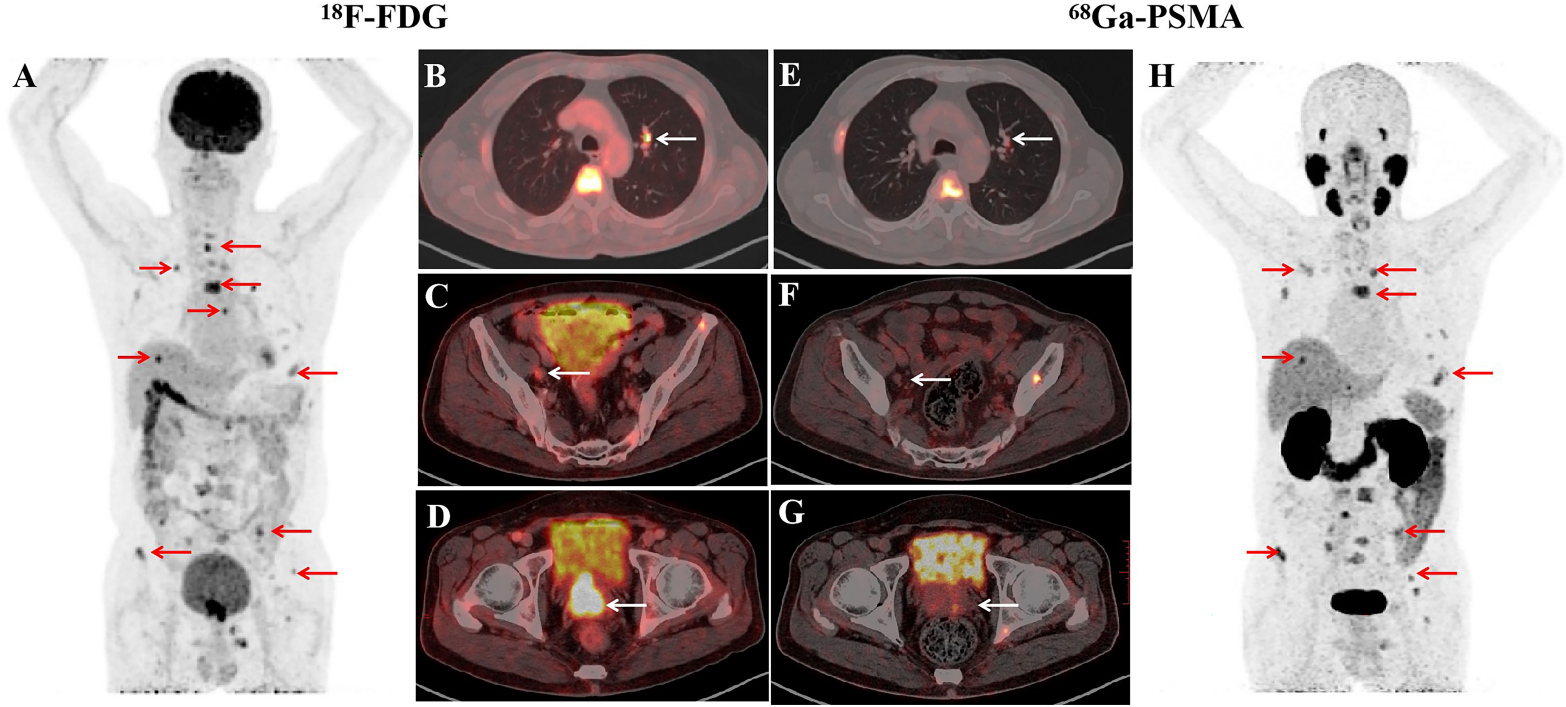
Imaging studies before the treatment of ADT combination with PARPi. In PET/CT images, PET maximum-intensity-projection image (MIP) **(A)**, Fused axial **(B–D)** showed intense diffuse accumulation of FDG in prostate mass (**D**, white arrow), lymph nodes (**C**, white arrow), pulmonary nodules (**B**, white arrow), bone lesions (**A**, red arrows). PSMA PET/CT images showed abnormal uptake of in bone lesions (**H**, red arrows), slight development of prostate mass (**G**, white arrow), lymph nodes (**F**, white arrow), pulmonary nodules (**E**, white arrow).

A man of 71-year was presented to the Department of Gastroenterology of our hospital with an elevated level of serum CEA (1296.5 ng/ml) and normal PSA (0.47 ng/ml) by regular cancer screening. Faecal occult blood test (FOBT) and gastrointestinal endoscopy showed no signs of gastric or colorectal cancer. Fluorodeoxyglucose positron emission tomography-computed tomography (FDG PET-CT) revealed a mass in the prostate, indicating prostate cancer with metastases lesions including lymph nodes adjacent to iliac vessels, bone as well as a mass of 0.9 cm in diameter at the upper lobe of left lung. A subsequent prostate-specific membrane antigen PET-CT (PSMA PET-CT) revealed the same mass and metastatic lesions, excepting for the lesion in left lung (**Figure 1**).

**Figure 2:**
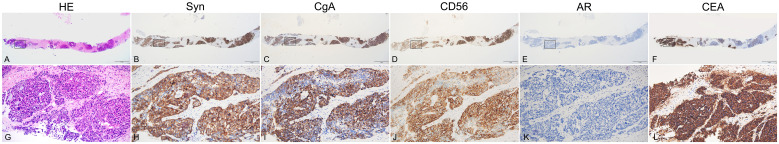
Biopsy specimen of the prostate. Hematoxylin-eosin (HE) and immunohistochemistry (IHC) staining of biopsy tissue from ONE same paraffin block. Tumor cells have round, ovoid, or spindled nuclei and scant cytoplasm with nesting, trabeculae or solid architectural patterns. Nuclear chromatin is finely granular and nucleoli are absent or inconspicuous **(A, G)**. In solid area, the tumor cells are positive for neuroendocrine markers SYN **(B, H)**, CgA **(C, I)**, CD56**(D, J)** and INSM1(not provided) but negative for AR expression **(E, K)**. As expected based on the elevated serum level of CEA, CEA is highly expressed **(F, L)**.

After radiological examination, a transperineal prostate systematic biopsy was performed. Hematoxylin-eosin (HE) and immunohistochemistry (IHC) revealed prostate carcinoma with negative staining of androgen receptor (AR) and strong expression of NE markers, including SYN, CgA, CD56 and INSM1 (**Figure 2**), indicating small cell neuroendocrine carcinoma (SCNEC) in 10 of 13 cores. The remaining 3 cores indicated mixed tumor of SCNEC and acinar adenocarcinoma (Gleason score of 4 + 5). Subsequent high-throughput sequencing revealed a germline mutation in BRCA2 (p.Y2541*). As for the lesion in left lung, no biopsy was performed because of the short diameter.

For the high level of CEA in serum, CEA expression in prostate biopsy tissue was also assayed. As demonstrated in (**Figures 2B, E**), a strong expression of CEA was detected in the slides of the same paraffin block where NE markers, including SYN (G, J), CgA (H, K), CD56(I, L) and INSM1(not provided) were also detected. Furthermore, to explore whether CEA could be used as a marker of diagnosis or monitoring in CEA-expressing NEPC, a commercial radioimmunoassay kit (Abbott CEA- RIA from Abbot Laboratories) was used. Collecting of expressed prostatic secretions (EPS) failed but the urine samples before and after prostatic massage were utilized. Compared with urine specimen before massage, the CEA concentrations from 12 ml urine specimens immediately post-massage increased sharply (137 ng/ml vs 5 ng/ml).

**Figure 3:**
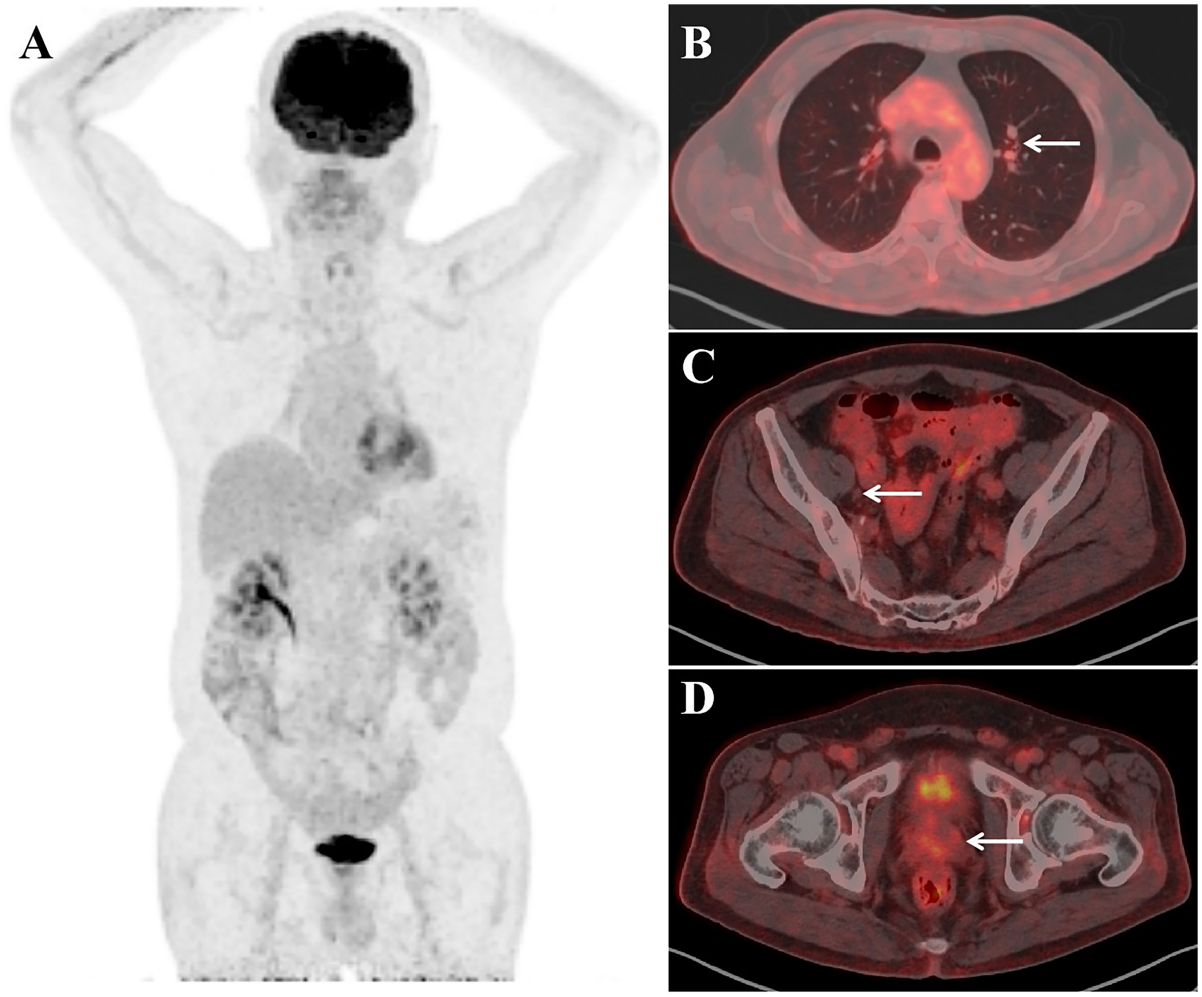
Imaging studies after the treatment of ADT combination with PARPi for two months. PET maximum-intensity-projection image (MIP) **(A)**, Fused axial **(B–D)** images showed no evidence of FDG–concentrating active disease foci.

After receiving a combination treatment of ADT and Olaparib (a PARPi) for 8 weeks, tumor regressed radiologically (**Figure 3**). Meanwhile the CEA levels in serum experienced decreases to 232.9 ng/ml and 74 ng/ml after treatment for 8 weeks and 12 weeks, respectively. Levels of CEA in about 10 ml of urine after prostatic massage also experienced sharp decreases (25.2ng/ml vs 37 ng/ml vs 137 ng/ml) 8 and 12 weeks, respectively (**Table 1**). This suggested that CEA in EPS or urine specimen post-prostatic massage might be a potential marker of NEPC.

**Table 1:** Level of prostatic tumor marker and CEA.

Time	tPSA (ng/ml)	NSE (ng/ml)	Serum CEA (ng/ml)	Urine CEA pre-massage (ng/ml)	Urine CEA post-massage (ng/ml)
Day 0	0.47	20.6	1296.5	5.0	137
4 weeks	< 0.09	10.61	918.4	Not available	Not available
8 weeks	< 0.09	10.68	232.9	3.7	37
12 weeks	< 0.09	11.76	74	3.22	25.2
16 weeks	< 0.09	10.12	23	0.71	2.4
20 weeks	< 0.09	11.0	47	Not available	Not available

## Discussion

NEPC, including t-NEPC and *de novo* NEPC, are rare diseases characterized by a poor prognosis, presenting with a low PSA and visceral metastases at diagnosis (4). These tumors can either express the conventional NE markers, including CgA, SYN, with or without CD56 expression, or even have complete lack of expression of any NE marker in about 10% of cases (3). However, the current available kits for measuring serum CgA has a low sensitivity of 2mg/L and were not widely used in clinic (5). SYN is a neuronal synaptic vesicle glycoprotein containing four transmembrane domains that form a hexameric channel or gap junction-like pore, not a secreted protein (6). These make NEPC a challenge for both the urologist and pathologist. An ideal fluid marker might facilitate the early screening or diagnosis of NEPC, especially *de novo* NEPC, which is rarer and more difficult to be diagnosed at early stage.

CEA belongs to a family of glycoproteins called carcinoembryonic antigen cell adhesion molecules (CEACAM) and is also known as CEACAM5 and CD66e (7). CEA is expressed in over 80% of colorectal cancers (CRC) and thought to be the preferred biomarker for *in vivo* CRC targeting (8). Recently, studies by Azra et al. demonstrated the expression of CEACAM5 in NEPC and supported CEACAM5 as a novel biomarkers or therapeutic targets for NEPC (9–13). As in the current case, CEA is expressed in NEPC tissue although not fully overlapping the zone where CgA, SYN and CD56 are expressed (**Figure 2**). Besides the current case, we reviewed 6 cases of NEPC we treated recently and detected CEA expression (by IHC) in 2 more patients, one *de novo* NEPC of large cell neuroendocrine carcinoma (LCNEC) and one t-NEPC.

Furthermore, to explore the feasibility of using CEA as a fluid marker for NEPC, we tried to assay the level of CEA in expressed prostatic secretion (EPS). However, for the difficulty of collecting of EPS in elderly men, especially those receiving hormonal therapy, urinary specimens after a prostatic massage were used as an alternative. In the current case, compared with urine before prostatic massage, we detected an increased level of CEA (5 ng/ml vs 137 ng/ml) in 12 ml of urine immediately after prostatic massage. A decreased level of CEA (25.2 ng/ml vs 137 ng/ml) in 10 ml of urine after prostatic massage was detected 3 months when tumor regressed radiologically (**Table 1**). This suggested that CEA in EPS or urine specimen post-prostatic massage might be a potential marker of NEPC.

As for the therapy, because of limited prospective clinical trial data, there is no standard strategies for managing NEPC, especially *de novo* NEPC. The mainstay of NEPC treatment is platinum-based chemotherapy and NEPC often responds to initial treatment with a median progression-free survival (PFS) of 2-8 months (14). However, after first-line chemotherapy, there is few effective treatment options available for NEPC, similar to small cell lung cancer (SCLC) (15).

So precise or personalized therapy based on genomic testing may be an attempt for patients of different subtypes of NEPC. A recent systematic review and meta-analysis demonstrated that the most frequently mutated gene in NEPC was TP53 (49.8%), and the prevalence of deleterious mutations in ATM/BRCA was 16.8% (16). Olaparib, a PARP inhibitor, is effective for patients with metastatic CRPC harboring BRCA gene alterations (17). Miyazawa et al. reported 2 cases of NEPC with BRCA2 mutation receiving Olaparib as second-line treatment and responding well (18). As in the current case, patient received Olaparib combined with ADT and Darolutamide as first-line treatment. Serum CEA and PSA reduced to 74 ng/ml and an undetectable level 3 months later, respectively.

This is the first report of a *de novo* NEPC presented with an elevated level of CEA, in which CEA was also detected in urine specimen post a prostatic massage. Treatment with ADT and PARPi targeting BRCA2 mutation resulted in partial regression in 3 months with a sharply decreased level of CEA in serum and urine. Although these trends of levels of CEA in serum and urine paralleled with clinic imaging, further clinical data is needed to verify whether CEA could be used as a fluid marker of screening and monitoring of NEPC.

## Data Availability

The raw data supporting the conclusions of this article will be made available by the authors, without undue reservation.
