# Supplementary material for: A case of de novo neuroendocrine prostate cancer presented with elevated level of serum CEA carrying BRCA2 mutation: case report and literature review
Source: Front Oncol. 2025 Jan 24;15:1508410. doi: 10.3389/fonc.2025.1508410 (PMC11802412; doi:10.3389/fonc.2025.1508410)
Supplement: Supplementary file 1 [file Table1.docx]

**Table. Results genetic variation test**

| **Types of variants** | **Counts of variants** | **Gene mutated** | |
| --- | --- | --- | --- |
| Pathogenic variants | 0 | Not detected | |
| Likely Pathogenic Variants | 2 | BRCA2 p.Y2541*(59.76%) | KRAS p.Q61H(38.37%) |
| Variants of Uncertain Significance | 14 | ARID2 p.Q995*(2.68%) | FANCD2 p.Q198H(30.88%) |
|  |  | HMCN1 p.V538L(5.83%) | IGFIR p.G34R(38.14%) |
|  |  | IRS2 p.P1091Rfs*15(2.24%) | JAK2 p.E461G(3.67%) |
|  |  | KMT2C p.K53Rfs*52(35.73%) | NOTCH1 p.R353P(10.54%) |
|  |  | PTCH1 p.R530I(40.90%) | RB1 c.1049+3A>G(55.88%) |
|  |  | TBX3 p.G129E(49.74%) | RB1^※^ (CN=1.21) |
|  |  | PRDM1^※^ (CN=1.16) | TP53^※^ (CN=1.08) |

^※^ Copy count reduction
